# Dual-tracer positron emission tomography/computed tomography as an imaging probe of *de novo* lipogenesis in preclinical models of hepatocellular carcinoma

**DOI:** 10.3389/fmed.2022.1008200

**Published:** 2022-09-27

**Authors:** Chin-Ho Tsao, Rong-Hong Jhou, Chien-Chih Ke, Chun-Wei Chang, Chi-Wei Chang, Bang-Hung Yang, Wen-Sheng Huang, Bing-Fu Shih, Ren-Shyan Liu

**Affiliations:** ^1^Department of Nuclear Medicine, Mackay Memorial Hospital, Taipei, Taiwan; ^2^Department of Medicine, Mackay Medical College, New Taipei City, Taiwan; ^3^Institute of Clinical Medicine, School of Medicine, National Yang Ming Chiao Tung University, Taipei, Taiwan; ^4^Department of Nuclear Medicine and National PET/Cyclotron Center, Taipei Veterans General Hospital, Taipei, Taiwan; ^5^Department of Medical Imaging and Radiological Sciences, Kaohsiung Medical University, Kaohsiung, Taiwan; ^6^Institute of Fisheries Science, National Taiwan University, Taipei, Taiwan; ^7^Department of Biomedical Imaging and Radiological Sciences, National Yang Ming Chiao Tung University, Taipei, Taiwan; ^8^Department of Nuclear Medicine, Cheng-Hsin General Hospital, Taipei, Taiwan; ^9^Molecular and Genetic Imaging Core, Animal Consortium, Taipei, Taiwan

**Keywords:** *de novo* lipogenesis, imaging probe, cancer metabolism, PET/CT, cancer therapy, lipogenesis-targeted therapy, hepatocellular carcinoma

## Abstract

**Background:**

*De novo* lipogenesis is upregulated in many cancers, and targeting it represents a metabolic approach to cancer treatment. However, the treatment response is unpredictable because lipogenic activity varies greatly among individual tumors, thereby necessitating the assessment of lipogenic activity before treatment. Here, we proposed an imaging probe, positron emission tomography/computed tomography (PET/CT) with dual tracers combining ^11^C-acetate and ^18^F-fluorodeoxyglucose (^18^F-FDG), to assess the lipogenic activity of hepatocellular carcinoma (HCC) and predict the response to lipogenesis-targeted therapy.

**Methods:**

We investigated the association between ^11^C-acetate/^18^F-FDG uptake and *de novo* lipogenesis in three HCC cell lines (from well-differentiated to poorly differentiated: HepG2, Hep3B, and SkHep1) by examining the expression of lipogenic enzymes: acetyl-CoA synthetase 2 (ACSS2), fatty acid synthase (FASN), and ATP citrate lyase (ACLY). The glycolysis level was determined through glycolytic enzymes: pyruvate dehydrogenase expression (PDH). On the basis of the findings of dual-tracer PET/CT, we evaluated the treatment response to a lipase inhibitor (orlistat) in cell culture experiments and xenograft mice.

**Results:**

Dual-tracer PET/CT revealed the lipogenic activity of various HCC cells, which was positively associated with ^11^C-acetate uptake and negatively associated with ^18^F-FDG uptake. This finding represents the negative association between ^11^C-acetate and ^18^F-FDG uptake. Because these two tracers revealed the lipogenic and glycolytic activity, respectively, which implies an antagonism between lipogenic metabolism and glucose metabolism in HCC. In addition, dual-tracer PET/CT not only revealed the lipogenic activity but also predicted the treatment response to lipogenesis-targeted therapy. For example, HepG2 xenografts with high ^11^C-acetate but low ^18^F-FDG uptake exhibited high lipogenic activity and responded well to orlistat treatment, whereas SkHep1 xenografts with low ^11^C-acetate but high ^18^F-FDG uptake exhibited lower lipogenic activity and poor response to orlistat.

**Conclusion:**

The proposed non-invasive dual-tracer PET/CT imaging can reveal the lipogenesis and glycolysis status of HCC, thus providing an ideal imaging probe for predicting the therapeutic response of HCC to lipogenesis-targeted therapy.

## Introduction

Targeting *de novo* lipogenesis represents a metabolic approach to cancer treatment (1–3) because lipogenesis is upregulated in various cancers (4, 5). This is achieved by inhibiting the lipogenic enzymes, such as fatty acid synthase (FASN) (5–7). However, the treatment response to lipogenesis inhibitors is highly variable (8) because of considerable intertumoral variation in lipogenic activity (9, 10). Therefore, non-invasive determination of the lipogenic activity of tumor cells before lipogenesis-targeted therapy is essential.

Imaging with ^11^C-acetate positron emission tomography/computed tomography (PET/CT) can assess the lipogenic activity and may thus help predict the response to lipogenesis-targeted therapy. ^11^C-acetate PET/CT has been used to evaluate the lipogenic metabolism in prostate cancer because ^11^C-acetate is an analog of lipogenic precursors and participates in the lipogenic pathway (11, 12). In addition, ^11^C-acetate PET imaging has a sensitivity of 87.3% in diagnosing hepatocellular carcinoma (HCC) (13), which can be increased to nearly 100% by using ^11^C-acetate in combination with ^18^F-fluorodeoxyglucose (^18^F-FDG). Although ^18^F-FDG PET/CT demonstrates glycolytic activity (14, 15), it is unclear whether the dual-tracer PET/CT combining ^11^C-acetate in combination with ^18^F-FDG is a better imaging probe for determining the lipogenic activity of HCC and predicting the therapeutic response to targeting lipogenesis.

The value of ^11^C-acetate and ^18^F-FDG PET imaging before and after molecular therapy was used to evaluate the prognosis of HCC patients in a recent study (16). The novelty of our study provides new insights of using dual-tracer PET/CT imaging to assess the lipogenic activity of various HCC cell lines with different differentiation states and to evaluate the therapeutic response of lipogenic inhibitor. Our findings revealed how ^11^C-acetate and ^18^F-FDG uptake varied with the expression of lipogenic and glycolytic enzymes, respectively, and demonstrated the association between lipogenesis and glycolysis in HCC. Dual-tracer PET/CT can not only provide an immediate metabolic status of HCC but may also serve as a more accurate imaging probe for *de novo* lipogenesis. By using the non-invasive dual-tracer imaging, we successfully predicted the therapeutic response to a lipogenesis inhibitor (orlistat) in HCC xenograft mice.

## Materials and methods

### Cell culture

We investigated three HCC cell lines (Bioresource Collection and Research Center Hsinchu, Taiwan) with various differentiation states: HepG2 (well-differentiated), Hep3B (moderately differentiated), and SkHep1 (poorly differentiated) (17). HepG2 and Hep3B cell lines were cultured in Dulbecco’s modified Eagle’s medium (Invitrogen/cat. no. 31800-012), and the SkHep1 cell line was cultured in minimum essential medium alpha (Invitrogen/cat. no. 12000-022). In all cell cultures, 10% fetal bovine serum (Hyclone) and 1% penicillin/streptomycin/glutamine cocktail (Invitrogen) were added to the growth media, and the cell lines were cultured at 37°C with 5% CO_2_ in humid incubators.

### Radiopharmaceutical preparation

The ^11^C-acetate for *in vivo* use was synthesized through the carbonation of Grignard reagent. ^11^C-carbon dioxide was reacted with 0.15 M methylmagnesium bromide and hydrolyzed with 0.4 M hydrochloric acid to be converted to ^11^C-acetate acid (18). All reaction steps were performed using a robotic system (Scanditronix Anatech RB III) controlled by a computer. The radiochemical purity was greater than 99%. ^18^F-FDG was synthesized as described in the literature (19). ^14^C-acetate for *in vitro* use was obtained from Blossom Biotechnologies.

### Cellular uptake of ^18^F-fluorodeoxyglucose and ^14^C-acetate

Aliquots of 1 μCi/500 μl of a radiotracer (^18^F-FDG or ^14^C-acetate) were added to each well containing preseeded cells cultured overnight in a 24-well culture plate at a density of 3 × 10^5^ cells/well. After 1-h incubation, the cellular uptake of radiotracers was terminated by rinsing the cells isolated from the supernatant in ice-cold phosphate-buffered saline (PBS). These cells were trypsinized and harvested. Next, the radioactivity of both (^18^F-FDG and ^14^C-acetate) harvested cells and the remaining supernatant was determined. Finally, all the cells were lysed, and the cellular proteins were extracted using CytoBuster Protein Extraction Reagent. The cellular uptake of ^18^F-FDG was determined as (radioactivity of cells)/[(radioactivity of supernatant) × (protein concentration)] by using the Cobra II Auto-Gamma Counter (PerkinElmer). For ^14^C-acetate uptake, we added scintillation fluid (Ultima Gold, PerkinElmer) to determine the radioactivity by using a Packard Tri-Carb LS β Counter (PerkinElmer). ^18^F-FDG and ^14^C-acetate uptake (gamma counter and beta counter, respectively) in cells is normalized by protein weight: counts per minute (CPM)/mg protein.

In the orlistat treatment group, 300 mM orlistat was added to the cell culture medium 2 h before the ^14^C-acetate uptake experiment. After 2 h, the cell uptake experiments continued, while orlistat remained in the culture medium.

### Animal model and *in vivo* micro-positron emission tomography/computed tomography imaging of ^18^F-fluorodeoxyglucose and ^11^C-acetate

The animal experiments were approved by the animal care and use committee of the institution. For the subcutaneous tumor models, HCC xenografts were established by inoculating approximately 10^7^ tumor cells into the shoulders of 7-week-old NOD/SCID mice (BioLASCO Taiwan). In total, 11 mice were inoculated with HepG2 cells and 10 with SkHep1 cells. When the volume of the inoculated tumor grew to approximately 100 mm^3^, the mice were injected with 500 μCi ^18^F-FDG or ^11^C-acetate through their tail vein and were subjected to whole-body micro-PET/CT imaging (FLEX Triumph preclinical PET/SPECT/CT scanner; TriFoil Imaging). Specifically, the mice were first subjected to CT imaging to acquire images of 256 × 256 pixels, at a current of 0.5 mA and voltage of 80 kVp, and then subjected to PET imaging for 20 min. The cone–beam reconstruction algorithm and 3D-ordered subset expectation maximization algorithm were applied to reconstruct the CT and PET images, respectively. All the reconstructed images were further analyzed using Amide (version 1.0.4; Crump Institute for Molecular Imaging, Los Angeles, CA, USA), a software for analyzing anatomical and functional volumetric medical imaging data in the Digital Imaging and Communications in Medicine standard (20). Specifically, Amide software was applied to represent the imaging with a color-based radiometric scale and to quantify the radioactivity in the regions of interest, including the tumors and thighs of the mice, and to calculate the tumor-to-muscle ratio (T/M ratio) based on percent intensity dose per gram (%ID/g) values (20).

### Quantitative real-time polymerase chain reaction

We conducted a quantitative real-time polymerase chain reaction (qPCR) to examine gene expression in HCC cells. First, we prepared the samples for qPCR by using TRIzol reagent (Invitrogen) to extract the total RNA from the tumor tissues. Then, the extracted RNA was reverse-transcribed into first-strand cDNA. By mixing the cDNA (40 ng) with SYBER Green PCR Master Mix (Applied Biosystems) and primers of target genes, qPCR was performed using a StepOnePlus Real-Time System (Applied Biosystems) under the following conditions: activation at 95°C for 20 s, followed by 40 cycles of 95°C for 1 s, and 60°C for 20 s. Finally, the qPCR results were normalized with respect to the expression of the housekeeping gene glyceraldehyde 3-phosphate dehydrogenase.

### Western blotting

Western blotting was used to extract lipogenic enzymes from the tumor cells by using the Bradford Protein Assay Kit (Bio-Rad). Specifically, 20 μg of bulk protein extracted from tumor cells with sample buffer (4 × SDS loading buffer: 10% glycerol, 62.5 mM Tris–HCl, pH: 6.8, 2% SDS, 65 mM DTT, and 0.05% bromophenol blue) was incubated in boiling water for 10 min. Through sodium dodecyl sulfate-polyacrylamide gel electrophoresis, the extracted protein was first transferred to polyvinylidene difluoride (PVDF) membranes, which were blocked with 5% (w/v) non-fat milk in PBS. Next, the transferred proteins on the PVDF membrane were reacted with primary antibodies, including anti-FASN (1:1,000, Genetex GTX109833), anti-ACSS2 (1:1,000, Genetex GTX30020), and anti-β-actin (1:1,000, Genetax GTX109639, as the internal control) at 4°C overnight. Next, the PVDF membranes were gently washed with TBST (50 mM Tris–HCl, pH: 7.4, 200 mM NaCl, 1% Tween-20) and incubated with HRPase-conjugated goat anti-rabbit IgG secondary antibodies (1:5,000; LI-COR Biosciences) at room temperature for 1 h. Finally, the chemiluminescent signals of the extracted proteins were examined using Western Lightning Plus-ECL and detected using the LAS 4000 mini-imaging system (Fujifilm).

### Incorporation of ^14^C-acetate into the lipid fraction

The activity of *de novo* lipogenesis in cultured HCC cells was assessed based on the proportion of cellular lipids derived from exogenous ^14^C-labeled acetate. First, preseeded tumor cells were incubated overnight in a 24-well culture plate at 6 × 10^4^ cells/well. Next, aliquots of 1 μCi/500 μl of ^14^C-acetate were added to each well and incubated for 1 h after the medium was removed and the cells were rinsed with PBS. Next, the cellular uptake of ^14^C-acetate was terminated by isolating the cells from the supernatant and rinsing them with ice-cold PBS. Subsequently, cellular lipids were extracted through centrifugation (1,000 rpm for 5 min) of the cells in the solution containing a mixture of 0.6 ml of MeOH (MeOH: H_2_O = 2.5:1) and 0.4 ml of CHCl_3_. Finally, ^14^C-acetate incorporated into the extracted lipids was determined using a Packard Tri-Carb LS β Counter (PerkinElmer) in scintillation solutions (PerkinElmer). ^14^C-acetate uptake in cells was normalized by protein weight (CPM/mg protein). In the orlistat treatment group, 300 mM orlistat was added to the cell culture medium 2 h before ^14^C-acetate incorporation into lipid experiments. After 2 h, the experiments were continued, with orlistat still in the culture medium.

### Cell cycle analysis

The response of the HCC cell cycle to lipogenesis-targeted treatment was examined. For this purpose, preseeded cells (6-well plates at 2 × 10^5^ cells/well) were treated with 150 μM orlistat dissolved in ethanol for 24 h and compared with the mock control treated with ethanol alone. Next, the orlistat treatment was terminated through addition of ice-cold 70% ethanol at −20°C for 1 h and washing of the cells with ice-cold PBS. Finally, the cells were stained with 40 μg/ml of propidium iodide reagent (BioVision) for 1 h at room temperature in the dark. Subsequently, the cell cycle phases were analyzed using a flow cytometer (FACS Calibur; BD Bioscience), which differentiated G0/G1, S, and G2/M phases according to the proportion of fluorescent areas in the cells (21).

### Tumor growth

To investigate the inhibitory effects of orlistat on HCC cells *in vivo*, we first established SkHep1 and HepG2 xenografts by inoculating 10^7^ tumor cells subcutaneously into the shoulder of ten 7-week-old NOD/SCID mice (five in the experimental group and five in the control group; BioLASCO Taiwan). The length and width of the tumors were measured every 2 days by using a caliper, and the tumor volume (mm^3^) was calculated as length × width^2^ × 0.52. When the length of the tumor reached 5 mm, orlistat was injected intraperitoneally into the mice at 240 mg/kg/day for 14 days. At the end of the study period, all mice were killed using carbon dioxide asphyxiation. All animal experiments were approved by the animal care and use committee of the institution.

### Statistical analysis

The statistical significance of the various experimental treatments was tested. Permutation tests were conducted for the treatments of orlistat (orlistat vs. control), and the Kruskal–Wallis test (non-parametric ANOVA) was performed to compare the three (or more)-level treatments (e.g., to evaluate the statistical differences between the three HCC cell lines).

## Results

**FIGURE 1:**
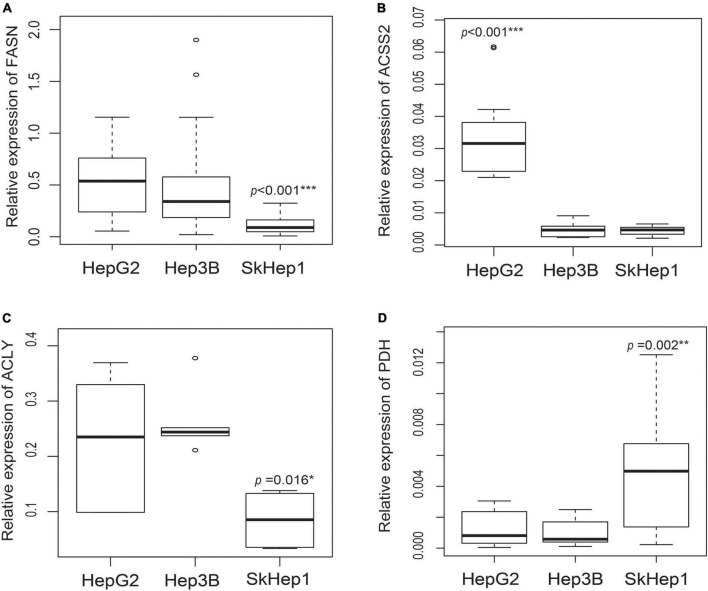
Gene expression of lipogenic enzymes and glycolysis-associated enzymes in three HCC cell lines. Gene expression analysis in three lipogenic enzymes through real-time PCR revealed that **(A)** FASN expression is significantly lower in SkHep1 cells than in Hep3B and HepG2 cells (Kruskal–Wallis test ***p < 0.001; *n* = 24 for each cell line), **(B)** ACSS2 expression is significantly higher in HepG2 cells than in Hep3B and SkHep1 cells (Kruskal–Wallis test ***p < 0.001; *n* = 12 for each cell line), and **(C)** ACLY expression (Kruskal–Wallis test *p = 0.016; *n* = 6 for each cell line) is significantly lower in SkHep1 cells than in Hep3B and HepG2 cells. **(D)** The expression of glycolysis-associated enzyme PDH in SkHep1 cells is significantly higher than that in Hep3B and HepG2 cells (Kruskal–Wallis test **p = 0.002; *n* = 15 for each cell line). We summarized all the measurements as box-plots that label 25, 50, and 75% quantiles in the boxes with whiskers presenting at most 1.5 × interquartile range.

The well-differentiated HepG2 cells exhibited high lipogenic activity, whereas the poorly differentiated SkHep1 cells exhibited low lipogenic activity. PCR and Western blotting indicated that HepG2 cells overexpressed three lipogenic enzymes: acetyl-CoA synthetase 2 (ACSS2), FASN, and ATP citrate lyase (ACLY) (Figure 1 and Supplementary Figure 1). By contrast, SkHep1 cells underexpressed the three lipogenic enzymes. In the moderately differentiated Hep3B cell line, lipogenesis was partially inhibited, and the expression of only ACSS2 was decreased. Thus, the results revealed a clear association between lipogenic activity and the differentiation status of HCC cell lines. Notably, the well-differentiated HepG2 cells exhibited not only high lipogenesis but also low glycolysis, as indicated by the underexpression of the glycolysis-associated enzyme pyruvate dehydrogenase (PDH) (Figure 1D). By contrast, SkHep1 cells exhibited not only low lipogenesis but also high glycolysis, as indicated by PDH overexpression.

**FIGURE 2:**
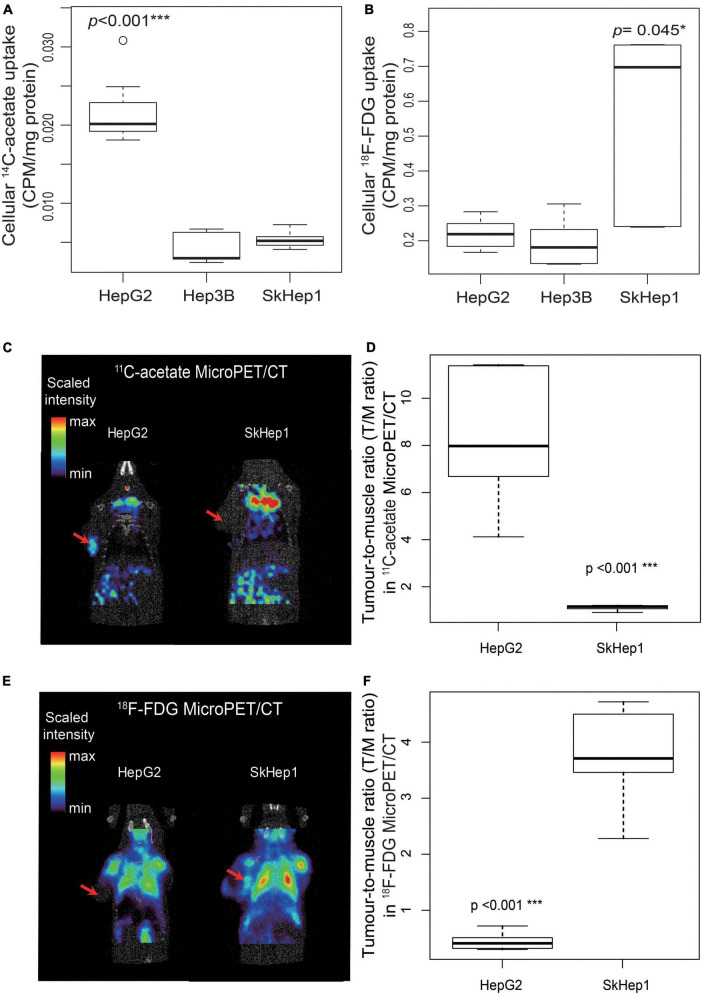
Dual-tracer uptake in three HCC cell lines *in vitro* and *in vivo*. *In vitro*, **(A)** ^14^C-acetate uptake in HepG2 cells is significantly higher than that in SkHep1 and Hep3B cells (Kruskal–Wallis test ***p < 0.001; *n* = 7, 10, and 10 for HepG2, Hep3B, and SKHep1, respectively). **(B)** ^18^F-FDG uptake in SkHep1 cells is significantly higher than that in HepG2 and Hep3B cells (Kruskal–Wallis test *p = 0.045; *n* = 6, 6, and 5 for HepG2, Hep3B, and SKHep1, respectively). *In vivo*, **(C)** ^11^C-acetate micro-PET imaging of HCC xenografts in nude mice, represented by a color-based radiometric scale, revealed that **(D)** ^11^C-acetate uptake assessed using the tumor-to-muscle ratio is significantly higher in HepG2 cells than in SkHep1 cells (**D**; permutation test ***p < 0.001; *n* = 6 and 5 for HepG2 and SkHep1, respectively); whereas **(E)** ^18^F-FDG micro-PET imaging of HCC xenografts revealed that **(F)** ^18^F-FDG uptake is significantly higher in SkHep1 cells than in HepG2 cells (**F**; permutation test ***p < 0.001; *n* = 5 for each cell line). The red arrows in dual-tracer micro-PET imaging in **C,E** indicate the location of the HCC xenografts. We summarized the measurements as box-plots that label 25, 50, and 75% quantiles in the boxes with whiskers presenting at most 1.5 × interquartile range.

**FIGURE 3:**
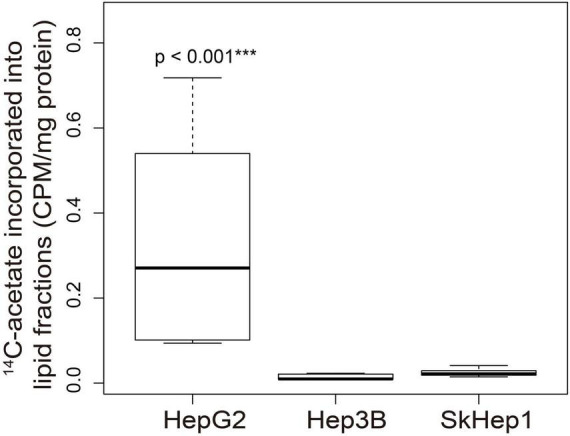
^14^C-acetate incorporated into cellular lipid fractions in three HCC cell lines. ^14^C-acetate was incorporated into the cellular lipid fraction in the three HCC cell lines. In the extracted lipid fraction, ^14^C-acetate uptake in HepG2 was significantly higher than that in Hep3B and SkHep1 (***p < 0.001; permutation test; *n* = 6, 9, and 9 for HepG2, Hep3B, and SkHep1, respectively), indicating that ^14^C-acetate was intensively accumulated in HepG2 cells and subsequently incorporated into cellular lipid fractions. ^14^C-acetate uptake (β counter) in cells normalized by protein weight (CPM/mg protein). We summarized the measurements as box-plots that label 25, 50, and 75% quantiles in the boxes with whiskers presenting at most 1.5 × interquartile range.

Dual-tracer PET/CT revealed high ^11^C-acetate uptake but low ^18^F-FDG uptake in the HepG2 cells, and low ^11^C-acetate uptake but high ^18^F-FDG uptake in the SkHep1 cells. In the *in vitro* culture experiments, acetate uptake was significantly higher but ^18^F-FDG uptake was significantly lower in HepG2 cells than in SkHep1 cells (Figures 2A,B, respectively). Similarly, *in vivo* micro-PET/CT imaging revealed significantly higher ^11^C-acetate accumulation and lower ^18^F-FDG accumulation in HepG2 xenografts than in SkHep1 xenografts (Figures 2C–F), which represents the negative association between ^11^C-acetate and ^18^F-FDG uptake. Considering that acetate uptake was correlated with lipogenic activity in HepG2 and SkHep1 cells, while ^18^F-FDG uptake was correlated with glycolytic activity (Figure 1 and Supplementary Figure 1), we concluded that there is an antagonism between lipogenic metabolism and glucose metabolism in HCC. The associations between lipogenic activity and acetate uptake were further confirmed with ^14^C-acetate addition experiments (Figure 3), in which the proportion of ^14^C-acetate-derived lipids in HepG2 cells was significantly higher than that in Hep3B and SkHep1 cells. Consequently, dual-tracer PET/CT combined with ^11^C-acetate and ^18^F-FDG are capable of revealing the lipogenesis and glycolysis of HCC, respectively, both *in vitro* and *in vivo* (Figure 2).

**FIGURE 4:**
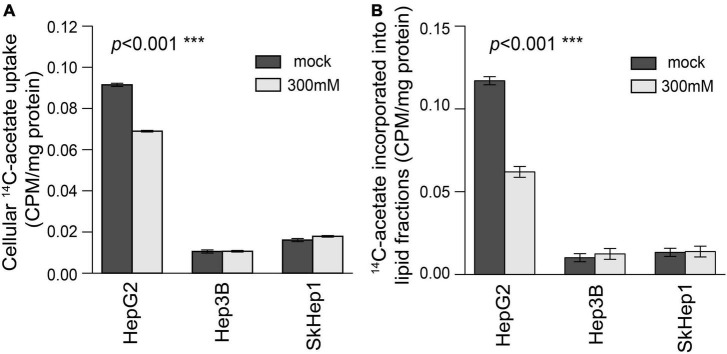
Treatment response to orlistat treatment assessed using ^14^C-acetate uptake and ^14^C-acetate incorporated lipid fractions in three HCC cell lines. In HepG2 cells, **(A)** ^14^C-acetate uptake and **(B)** ^14^C-acetate incorporated lipid fractions were significantly inhibited by orlistat treatment (labeled by light gray) compared with mock treatment (labeled by dark gray) (permutation test ***p < 0.001 in both **A,B**; *n* = 6 for each bar). However, orlistat-induced inhibition was not significant in Hep3B and SkHep1 (permutation test *p* = 0.236 and 0.630, respectively; *n* = 6 for each bar). ^14^C-acetate uptake (β counter) in cells normalized by protein weight (CPM/mg protein). We summarized these measurements as bar plots that label the averaged values with error bars presenting the standard deviation (±1 SD) computed from six replicates for each treatment (*n* = 6 for each bar).

**FIGURE 5:**
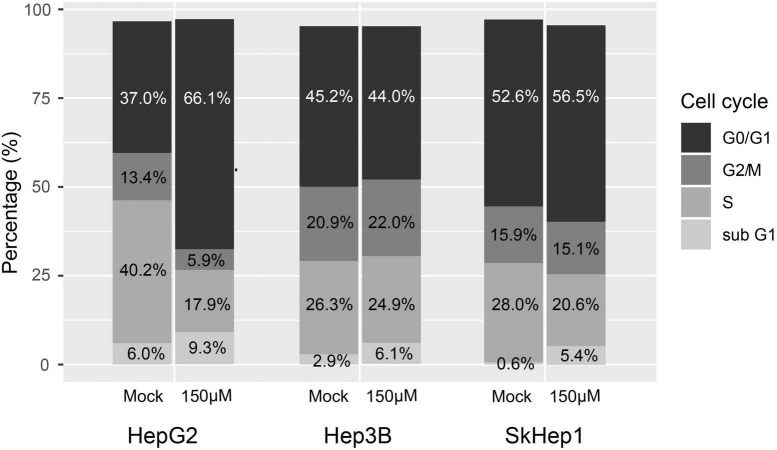
Treatment response to orlistat assessed using cell cycle analysis with flow cytometry in three HCC cell lines. HepG2 cells in the S/G2/M cell cycle were obviously reduced after orlistat treatment compared with mock treatment, whereas no apparent change was observed in the cell cycle in Hep3B and SkHep1 cells. The proportion was determined by counting 2 × 10^5^ cells for each treatment (*n* = 2 × 10^5^).

**FIGURE 6:**
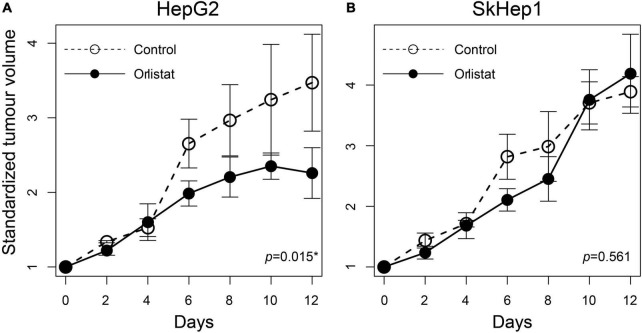
Treatment response to orlistat in HCC xenografts assessed using tumor growth rates in nude mice. The tumor doubling time was significantly reduced in **(A)** orlistat-treated HepG2 xenograft mice compared with controls (*p = 0.015; *n* = 2 and 3 xenografts for control and orlistat treatment, respectively), whereas no significant difference was observed in **(B)** SkHep1 xenografts (*p* = 0.561; *n* = 3 xenografts for each treatment). The open and solid circles are the averaged standardized tumor volumes for control and orlistat treatment, respectively. The averaged tumor volume as well as its error bar presenting the uncertainty (±1 SD) was calculated from the replicates observed at different times.

Lipogenic activity was used to determine the response of HCC to orlistat treatment *in vitro* and *in vivo*. In the *in vitro* experiments, HepG2 cells with high lipogenic activity (Figure 1 and Supplementary Figure 1) had a good response to orlistat, which was indicated by the significant reduction in the uptake of ^14^C-acetate and in the production of ^14^C-acetate-derived lipids (Figure 4). In addition, orlistat inhibited the proliferation of HepG2 cells, which significantly reduced the number of cells in the S/G2/M cell cycle phase (Figure 5). By contrast, the other two cell lines, Hep3B and SkHep1, with decreased lipogenic activity, showed a poor response to orlistat treatment and no significant reduction in proliferation. Similar to *in vitro* findings, orlistat significantly inhibited tumor growth in HepG2 xenografts, but not in SkHep1 xenografts (Figure 6). That is, cell lines with high lipogenic activity responded well to lipogenesis-targeted treatment with orlistat. As mentioned earlier, lipogenic activity in HCC cells can be indicated by dual-tracer PET/CT imaging combined with ^11^C-acetate and ^18^F-FDG; our results verify that dual-tracer PET/CT imaging can not only assess lipogenic activity but also predict the response to lipogenesis-targeted therapy in a non-invasive manner.

## Discussion

^14^C-acetate uptake revealed the activity of *de novo* lipogenesis involving multiple lipogenic enzymes in HCC, whereas the expression of individual lipogenic enzymes (e.g., FASN) revealed only partial information on lipogenesis. Although ^14^C-acetate uptake was positively associated with FASN expression in HepG2 and SkHep1 cells (Figures 1, 2), Hep3B cells exhibited a discrepancy between ^14^C-acetate uptake (low) and FASN expression (high), consistent with a previous study on HCC (22). Despite Hep3B cells highly expressing FASN, the reduction in ^14^C-acetate uptake more accurately revealed the reduction in lipogenesis (Figures 2, 3) and better predicted its poor response to antilipogenic therapy with orlistat (Figure 4). ^14^C-acetate can reveal lipogenic activity because it is involved in multiple steps of the lipogenic pathway (11, 12), whereas FASN is involved in only one step of the lipogenic pathway. Consequently, ^14^C-acetate uptake level provides a more comprehensive assessment of lipogenic activity than individual lipogenic enzymes do, whereas more detailed analysis is still required for examining the incorporation of ^14^C-acetate in the metabolism of various lipid classes.

Compared with the single tracer of ^14^C-acetate, dual tracers combining ^14^C-acetate with ^18^F-FDG more accurately assess lipogenic activities across various HCC cells. For instance, in the absence of ^18^F-FDG, only ^14^C-acetate uptake failed to distinguish the differences in lipogenic activity between Hep3B cells and SkHep1 (Figure 2A) because they had similar acetate uptakes. Nonetheless, the addition of ^18^F-FDG imaging can help distinguish the lipogenic activity between Hep3B and SkHep1 cells because SkHep1 cells have high glycolytic activity coupled with low lipogenic activity (Figures 1, 2B); a biochemical connection between glycolysis and lipogenic metabolism in HCC has been reported (23). Therefore, to better distinguish lipogenic activity among the various HCC cell lines, use of dual tracers is essential. Moreover, a comprehensive *in vivo* imaging analyses before and after lipogenesis-targeted therapy, including more types of HCC cell lines, are warranted to corroborate our findings. In addition, with the technical progress of new generation PET/CT instruments, the effective radiation dose of dual-tracer PET/CT has been substantially reduced to 14.3 mSv to avoid the radiation burden of HCC patients, compared with diagnostic abdominal CT with the effective radiation dose of 20 mSv (24–26). We suggest that the proposed dual-tracer PET imaging can track cancer metabolic changes and provide a clearer picture for designing suitable and individualized treatment strategies.

In addition to lipogenic and glycolytic metabolism, dual-tracer PET/CT imaging can reveal other metabolic pathways of HCC cells, which can be used to predict the prognosis of patients with HCC. In our study, dual PET/CT imaging in well-differentiated HepG2 cells exhibited high ^11^C-acetate accumulation and low ^18^F-FDG. Increased ^11^C-acetate uptake might indicate not only increased lipogenesis (Figures 1, 2) but also increased oxidative phosphorylation (27), in which catalyzed ^11^C-acetate (e.g., for producing acetyl-CoA) plays critical roles in both lipogenesis and oxidative phosphorylation metabolic pathways. However, when ^11^C-acetate uptake is reduced (e.g., in poorly differentiated SkHep1 in Figures 1, 2), metabolic status may shift from active lipogenesis and oxidative phosphorylation, respectively, to increased demand for exogenous lipids (overexpression of lipid transporters such as lipoprotein lipase, LPL) (28, 29) and exogenous glucose (overexpression of glucose transporter, e.g., glucose transporter 1, GLUT1 for anaerobic glycolysis) (30, 31). Because both LPL and GLUT1 are considered poor prognosis factors of HCC (29, 31, 32), dual-tracer PET/CT imaging might be used to unveil the metabolic status of HCC to predict the prognosis of patients with HCC. Nevertheless, the application of dual-tracer imaging to explore cancer metabolism and associated treatments requires more comprehensive *in vivo* imaging and *in vitro* experimental data that target gene and protein expression of various enzymes involved in different stages of metabolic pathways in future works.

Cholesterol is a major lipid component of cell membrane and lipoproteins. Cholesterol metabolism provides the prognostic signature for HCC (33). There is a functional crosstalk in hepatocarcinogenesis between cholesterol biosynthesis and *de novo* lipogenesis, sharing the same precursor of acetyl-CoA (34, 35). Thus, ^11^C-acetate tracer can be also involved in cholesterol metabolism. However, the relative proportions of acetyl-CoA involved in cholesterol metabolism and *de novo* lipogenesis in HCC remain unclear (36) and require more detailed investigations in the future.

Dual-tracer imaging can serve as a non-invasive method for selecting patients with HCC with high lipogenic activity for lipogenesis-targeted therapy, thereby facilitating the development of commercial drugs (Supplementary Figure 2). Currently, antilipogenic drugs targeting lipogenic enzymes (2, 37, 38) and transcription factors (39–41) are rapidly being developed. However, none of these drugs have completed clinical human trials or entered commercial production. One possible reason is the lack of treatment guidance for selecting patients with HCC who respond well to these drugs. Our dual-tracer PET/CT imaging can reveal the lipid metabolism status of HCC cells to provide image guidance for the selection of patients with HCC with high lipogenic activity. For example, patients with HCC with high ^11^C-acetate but low ^18^F-FDG uptake on dual-tracer PET/CT exhibit high lipogenic activity and may respond well to lipogenesis inhibitors (Figures 4, 6). By contrast, patients with low ^11^C-acetate but high ^18^F-FDG uptake exhibit low lipogenic activity and may respond poorly to lipogenesis inhibitors, although alternative therapies that target exogenous lipid transporters (e.g., LPL) may elicit a good response (42). This imaging-based treatment guidance must be validated in more detailed analyses and clinical trials. Our proposed dual-tracer PET/CT imaging opens up a promising research direction to reveal the lipogenic metabolism status of HCC in a non-invasive manner. Eventually, the proposed imaging probe might guide the future development of lipogenesis-targeted drugs through appropriate patient selection.

## Conclusion

Our proposed non-invasive dual-tracer PET/CT combining ^11^C-acetate with ^18^F-FDG is an imaging probe that can effectively reveal the lipogenic activity of HCC cells and predict their response to lipogenesis-targeted therapy.

## Data availability statement

The original contributions presented in this study are included in the article/Supplementary material, further inquiries can be directed to the corresponding authors.

## Ethics statement

This study was performed in accordance with a plan that was reviewed and approved by the Ethics Committee for Animal Experiments of National Yang Ming Chiao Tung University.

## Author contributions

C-HT and R-SL conceived and designed the study. R-HJ and C-CK performed the experiments. Chi-WC prepared the radiopharmaceuticals. C-HT and Chu-WC analyzed the data. C-HT and R-SL wrote the manuscript, with critical comments from the co-authors. All authors contributed to the article and approved the submitted version.

## Supplementary material

Supplementary Figure 1 Protein expression in two lipogenic enzymes, FASN and ACSS2, by Western blotting in three HCC cell lines. Our analysis revealed that **(A)** FASN expression was lower in SkHep1 cells than in Hep3B and HepG2 cells and **(B)** ACSS2 expression in HepG2 cells was higher than that in Hep3B and SkHep1 cells. Protein expression level is quantified in a relative sense with respect to protein expression of housekeeping gene, β-actin.

Supplementary Figure 2 Schematic diagram of *de novo* lipogenesis. The diagram presents biochemical processes that critically affects lipid synthesis *via* the key enzymes for possible drug targets (red box; FASN, ACLY, and ACSS2 were analyzed in this study). In this study, we examined the treatment response of HCC to Orlistate treatment (FASN inhibitor).
