# Supplementary figures and images for: Dual-tracer positron emission tomography/computed tomography as an imaging probe of de novo lipogenesis in preclinical models of hepatocellular carcinoma
Source: Front Med (Lausanne). 2022 Sep 27;9:1008200. doi: 10.3389/fmed.2022.1008200 (PMC9551611; doi:10.3389/fmed.2022.1008200)

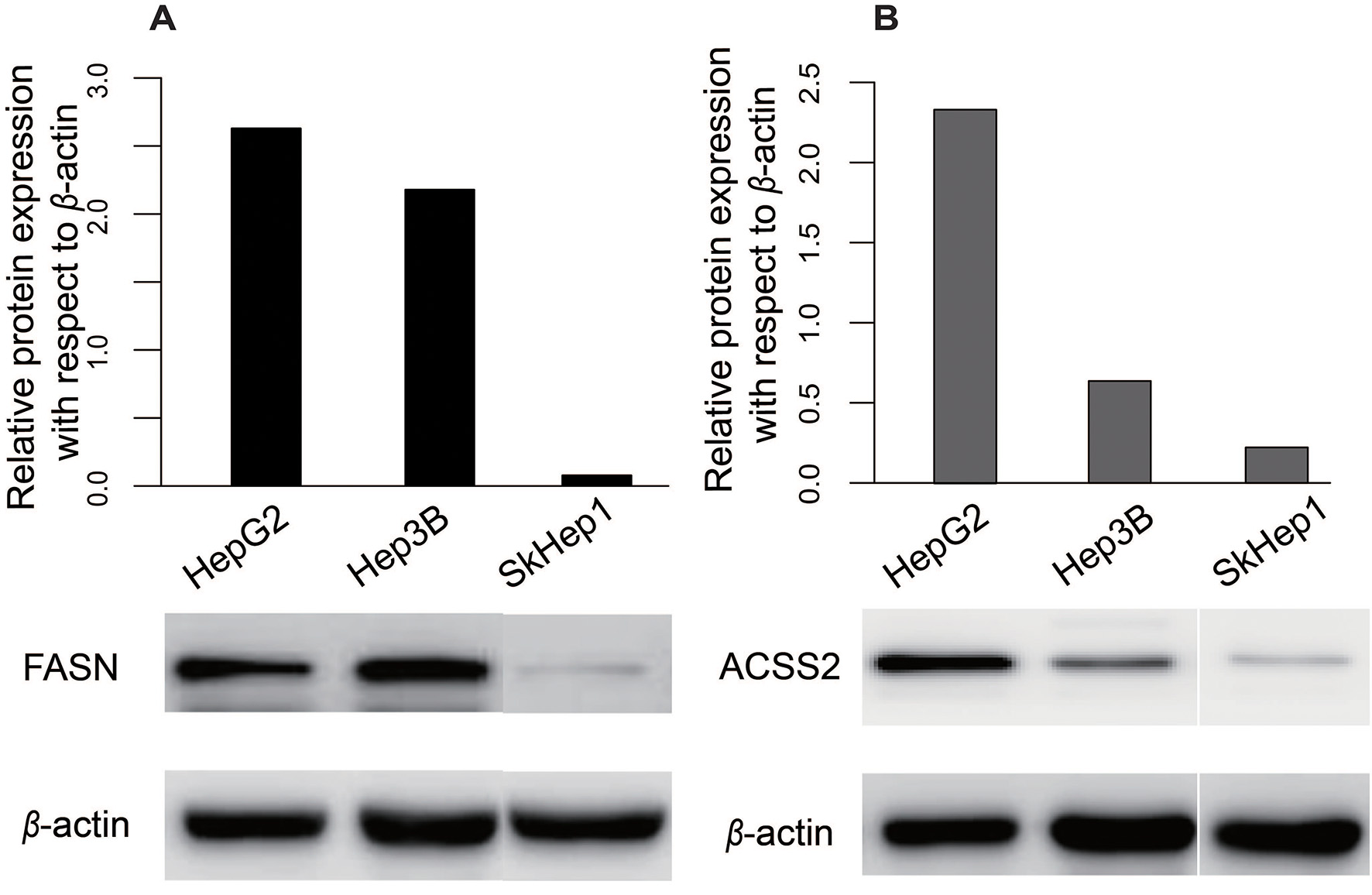

Supplement: Supplementary Figure 1 — Protein expression in two lipogenic enzymes, FASN and ACSS2, by Western blotting in three HCC cell lines. Our analysis revealed that (A) FASN expression was lower in SkHep1 cells than in Hep3B and HepG2 cells and (B) ACSS2 expression in HepG2 cells was higher than that in Hep3B and SkHep1 cells. Protein expression level is quantified in a relative sense with respect to protein expression of housekeeping gene, β-actin. [file Image_1.JPEG]

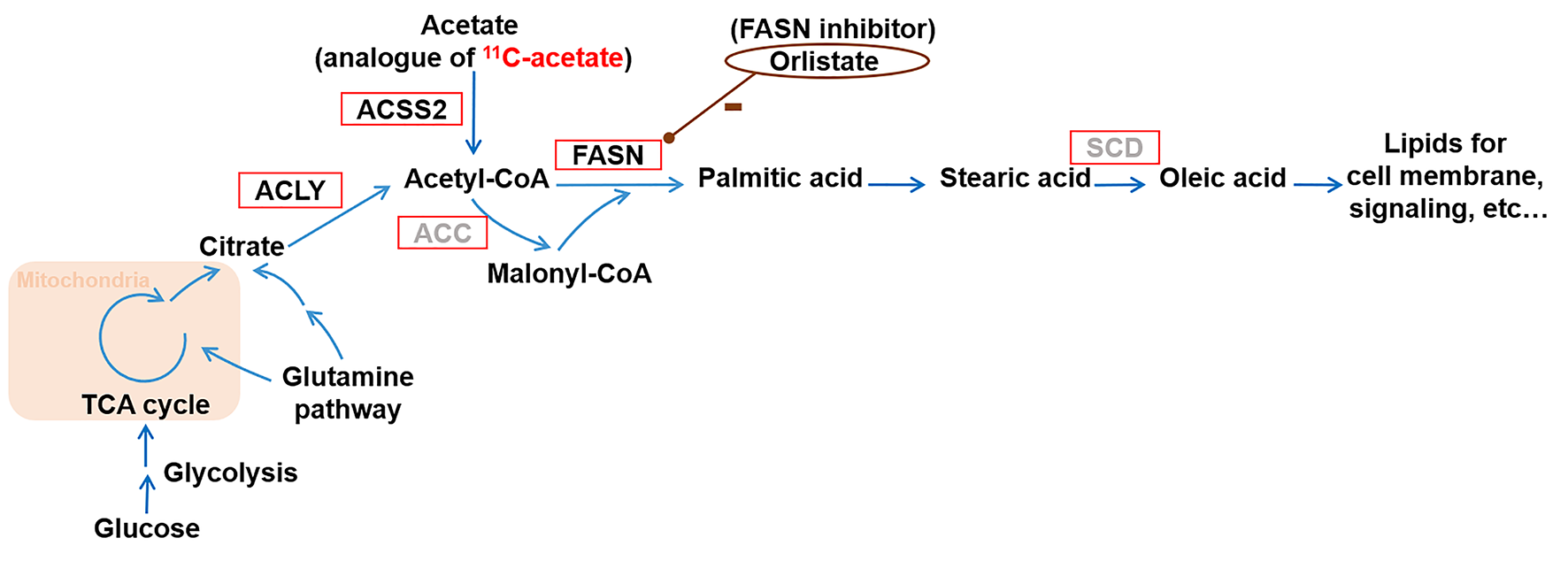

Supplement: Supplementary Figure 2 — Schematic diagram of de novo lipogenesis. The diagram presents biochemical processes that critically affects lipid synthesis via the key enzymes for possible drug targets (red box; FASN, ACLY, and ACSS2 were analyzed in this study). In this study, we examined the treatment response of HCC to Orlistate treatment (FASN inhibitor). [file Image_2.PNG]
